# Females’ Peer Influence and Support for Adolescent Males Receiving Voluntary Medical Male Circumcision Services

**DOI:** 10.1093/cid/cix1057

**Published:** 2018-04-03

**Authors:** Michelle R Kaufman, Kim H Dam, Kriti Sharma, Lynn M Van Lith, Karin Hatzold, Arik V Marcell, Webster Mavhu, Catherine Kahabuka, Lusanda Mahlasela, Eshan U Patel, Emmanuel Njeuhmeli, Kim Seifert Ahanda, Getrude Ncube, Gissenge Lija, Collen Bonnecwe, Aaron A R Tobian

**Affiliations:** 1 Johns Hopkins Bloomberg School of Public Health, Baltimore, Maryland; 2 Johns Hopkins Center for Communication Programs, Baltimore, Maryland; 3 Population Services International, Harare, Zimbabwe; 4 Department of Pediatrics, Johns Hopkins University School of Medicine, Baltimore, Maryland; 5 Centre for Sexual Health and HIV/AIDS Research, Harare, Zimbabwe; 6 CSK Research Solutions, Ltd, Dar es Salaam, Tanzania; 7 Centre for Communication Impact, Pretoria, South Africa; 8 Department of Pathology, Johns Hopkins University School of Medicine, Baltimore, Maryland; 9 Office of HIV/AIDS, Global Health Bureau, United States Agency for International Development, Washington, District of Columbia; 10 Ministry of Health and Child Care, Harare, Zimbabwe; 11 Ministry of Health, Community Development, Gender, Elderly and Children, Dar es Salaam, Tanzania; 12 National Department of Health, Pretoria, South Africa

**Keywords:** adolescents, voluntary medical male circumcision, HIV prevention, females, sub-Saharan Africa

## Abstract

**Background:**

While female involvement in voluntary medical male circumcision (VMMC) has been studied among adults, little is known about the influence of adolescent females on their male counterparts. This study explored adolescent females’ involvement in VMMC decision making and the postoperative wound healing process in South Africa, Tanzania, and Zimbabwe.

**Methods:**

Across 3 countries, 12 focus group discussions were conducted with a total of 90 adolescent females (aged 16–19 years). Individual in-depth interviews were conducted 6–10 weeks post-VMMC with 92 adolescent males (aged 10–19 years). Transcribed and translated qualitative data were coded into categories and subcategories by 2 independent coders.

**Results:**

Adolescent female participants reported being supportive of male peers’ decisions to seek VMMC, with the caveat that some thought VMMC gives males a chance to be promiscuous. Regardless, females from all countries expressed preference for circumcised over uncircumcised sexual partners. Adolescent females believed VMMC to be beneficial for the sexual health of both partners, viewed males with a circumcised penis as more attractive than uncircumcised males, used their romantic relationships with males or the potential for sex as leveraging points to convince males to become circumcised, and demonstrated supportive attitudes in the wound-healing period. Interviews with males confirmed that encouragement from females was a motivating factor in seeking VMMC.

**Conclusions:**

Adolescent female participants played a role in convincing young males to seek VMMC and remained supportive of the decision postprocedure. Programs aiming to increase uptake of VMMC and other health-related initiatives for adolescent males should consider the perspective and influence of adolescent females.

Voluntary medical male circumcision (VMMC) reduces the risk of acquiring human immunodeficiency virus (HIV), human papillomavirus, and herpes simplex virus type 2 among men [1–10], and *Trichomonas vaginalis*, bacterial vaginosis, and human papillomavirus among female partners [11–13]. Women also benefit indirectly from the expansion of VMMC services because the probability of encountering an HIV-infected male partner gradually declines with programmatic scale-up [14]. Mathematical models have also shown a 46% long-term reduction in male-to-female HIV transmission due to reduced male susceptibility following VMMC [15]. A recent study of women in KwaZulu Natal, South Africa, showed that those with circumcised partners had a 30% lower likelihood of having HIV and were less likely to have herpes simplex virus type 2 [16].

While female involvement in VMMC decision making has been studied among adults [17–19], little is known regarding the influence of female peers on adolescent VMMC clients. Research on adolescents in sub-Saharan Africa shows that peers have a large impact on adolescent sexual and reproductive health behaviors [20–26]. Understanding adolescent females’ level of influence on adolescent VMMC uptake may be important for programmatic scale-up and sustainability. This study utilized focus groups with female adolescents aged 16–19 years and in-depth interviews (IDIs) with male adolescents aged 10–19 years to explore the influence and support of adolescent females in the decision-making and healing process for male peers and sexual partners receiving VMMC across 3 countries: South Africa, Tanzania, and Zimbabwe.

## METHODS

### Female Participants

Focus group discussions (FGDs; 6–10 participants in each) were conducted with 90 female adolescents aged 16–19 years in South Africa, Tanzania, and Zimbabwe (4 FGDs per country). Female adolescent participants were recruited from the communities in which VMMC clinics were located, and male adolescent participants were recruited from 4 VMMC sites per country, as previously described [27]. VMMC community mobilizers (who encouraged male adolescents to be circumcised and were familiar with adolescents in the area) and/or trained research coordinators recruited female participants at youth groups, youth-gathering locations, and schools. Female participants were not necessarily sexual partners of VMMC clients.

FGDs with female adolescents focused on their opinions and perceptions of VMMC and examples of how female adolescents try to convince their boyfriends to seek VMMC. They were asked about their knowledge of VMMC’s impact on HIV and on hygiene, as well as their views about VMMC’s influence on relationships in general and on sexual behaviors. Male adolescents were asked about their experiences in disclosing their VMMC status and/or discussing the procedure experience and perceptions with their female adolescent peers.

### Male Participants

IDIs were conducted with 92 male adolescents (aged 10–19 years) 6–10 weeks post-VMMC procedure (South Africa, n = 36; Tanzania, n = 36; Zimbabwe, n = 20). This timeframe was selected to allow for completion of the full post-VMMC healing period and any follow-up appointments. Male participants were recruited at the health facility by trained research coordinators working with VMMC mobilizers on the day of their procedure or VMMC providers during their follow-up appointment, as previously described [28].

### Ethics and General Procedures

The Human Sciences Research Council in South Africa, Tanzania National Institute for Medical Research, Medical Research Council of Zimbabwe, and Johns Hopkins Bloomberg School of Public Health Institutional Review Board approved the study prior to data collection. Parental permission was obtained for participants <18 years of age, and assent/consent was obtained for all participants.

Data were collected during June–September 2015 in Tanzania, August–December 2015 in Zimbabwe, and February–June 2016 in South Africa. Interviews were conducted in private settings by local, trained facilitators in English or local languages: Sesotho, isiZulu, or isiSwati in South Africa; kiSwahili in Tanzania; Shona or Ndebele in Zimbabwe. Interviews were audio recorded, transcribed, and translated into English. 

### Analyses

Two coders independently coded transcripts using Atlas.ti software. The 2-step coding process included an initial independent read-through of all transcripts by each coder to develop a coding scheme. The coders then discussed any differences in the scheme until a consensus was met. Research staff double-coded all transcripts, and when further content analysis arose, coders generated themes and subthemes within predetermined areas of inquiry in the semistructured interview guides. Coders compared all applied codes and discussed discrepancies until an agreement was reached. In the rare event that an individual code resulted in no agreement between coders, the primary investigator made a final determination.

## RESULTS

Table 1 shows the participant demographics overall and by country. Table 2 displays the themes and subthemes that arose from the adolescent female and male client perspectives.

**Table 1.** Study Participant Demographics by Country

Characteristic	Total	South Africa	Tanzania	Zimbabwe
**Female adolescents (FGDs**)	(N = 90)	(n = 28)	(n = 28)	(n = 34)
Age, y				
16–17	37 (41.1)	13 (46.4)	10 (35.7)	14 (41.2)
18–19	53 (58.9)	15 (53.6)	18 (64.3)	20 (58.8)
Mean (SD)	17.6 (1.2)	17.5 (1.2)	17.7 (1.2)	17.5 (1.2)
Setting				
Urban	54 (60.0)	8 (28.6)	20 (71.4)	26 (76.5)
Periurban	21 (23.3)	13 (46.4)	8 (28.6)	0 (0.0)
Rural	15 (16.7)	7 (25.0)	0 (0.0)	8 (23.5)
**Male adolescents (IDIs**)	(N = 92)	(n = 36)	(n = 36)	(n = 20)
Age, y				
10–14	49 (53.3)	28 (77.8)	15 (41.7)	6 (30.0)
15–19	43 (46.7)	8 (22.2)	21 (58.3)	14 (70.0)
Mean (SD)	14.5 (2.9)	13.4 (2.3)	15.1 (3.4)	15.5 (2.3)
Setting				
Urban	55 (59.8)	9 (25.0)	31(86.1)	15 (75.0)
Periurban	14 (15.2)	9 (25.0)	5 (13.9)	0 (0.0)
Rural	23 (25.0)	18 (50.0)	0 (0.0)	5 (25.0)

Data are presented as n (%) unless otherwise indicated.

Abbreviations: FGDs, focus group discussions; IDIs, in-depth interviews; SD, standard deviation.

**Table 2.** Themes by Participant Perspective and Subthemes Within Each

Themes	Participant Perspective	Subthemes
Female adolescents’ acceptance of VMMC		
	Females	Many female peers supported, respected, and preferred males who underwent VMMC.
	Females	Female participants found VMMC to be a positive step towards healthier sexual relations for both men and women.
	Females	Some female participants saw VMMC as a driver for promiscuous behavior.
Females’ role in VMMC decision making		
	Females and males	Male adolescents were reluctant to discuss VMMC with female peers and platonic friends.
	Females and males	Romantic and/or sexual partners influence the VMMC decision-making process for males.
	Females	Many females leverage their relationship status to encourage males to seek VMMC.
	Females and males	Females’ preference for dating or having sex with circumcised males is a factor in males’ decision to undergo VMMC.
	Females and males	Males were perceived as more attractive and confident following VMMC, which was viewed by females as a positive thing, unless they used that new confidence to be promiscuous.
Female support after VMMC		
	Males	Adolescent males felt supported by female peers, partners, and family members after VMMC.

Abbreviation: VMMC, voluntary medical male circumcision.

### Female Adolescents’ Acceptance of VMMC

#### Female Beliefs Regarding VMMC Benefits

Female participants believed VMMC is mutually beneficial for healthier sexual relations and considered it a modern prerequisite to date a male. When female participants were prompted regarding the benefits of VMMC, most mentioned the protection against HIV infection, sexually transmitted infections, and cervical cancer in the female sexual partners of circumcised males.

It reduces the chances of contracting diseases like HIV, cancer …especially HIV—that is the one we dread most as young people who are still growing up. If you have a circumcised partner, you know that you are on the “safe side.” (Female, age 19, Harare, Zimbabwe)

Adolescent females from all 3 countries expressed their overall preference for circumcised sexual partners.

They [circumcised males] would have done a good thing because they would have protected themselves from sexually transmitted diseases. He would have helped me ... He would have helped himself and me as well in terms of preventing sexually transmitted diseases. So it is good to be circumcised. (Female, age 18, Mutare, Zimbabwe)

#### Female Support of VMMC

In general, adolescent female participants were supportive of male peers’ decisions to seek VMMC. Many female participants in Tanzania and Zimbabwe shared that they respected young men who underwent VMMC; they viewed it as a brave decision to undergo the procedure to improve both partners’ health.

I think it is a must [to receive VMMC], not a matter of choice, of willingness. The world we live in today is different because there are so many diseases, unlike how the situation was back in the days when people could live without being circumcised and still not risk getting diseases. So because circumcision helps in reducing the risk of diseases, I think it is a must for every male to be circumcised. (Female, age 19, Makambako, Tanzania)

While many females shared that they admired males who underwent the procedure, some were skeptical and believed males would use their circumcised status as an opportunity for promiscuity.

I don’t admire him [a circumcised male] at all. He is the same as before even though he is circumcised. He will take advantage now that he is safe and do crazy things. (Female, age 16, Ermelo, South Africa)

### Females’ Role in VMMC Decision Making

#### Discomfort Talking About VMMC With Female Peers

Male adolescents reported that VMMC was a personal matter not to be discussed with others. Most adolescent males across all countries reported they rarely considered the opinions of their female peers and platonic friends when deciding to seek VMMC. Some males even reported not feeling comfortable talking with female friends about their VMMC status, citing shyness and fear of being ridiculed or mocked.

There aren’t any [girls my age that know I am circumcised] because … it is actually embarrassing [laughs] … because I am a boy and she is a girl. (Male, age 14, Mutare, Zimbabwe)

This fear of potential embarrassment or ridicule from disclosing VMMC status was observed in all countries. Female adolescents also explained that their male friends often did not feel comfortable talking about VMMC with them.

Very few boys can talk about their circumcision experience with girls; they talk about it among themselves. If they talk about it with girls, then they must be very confident. (Female, age 18, Makambako, Tanzania)

#### Comfort Talking About VMMC With Female Partners

In contrast to male adolescents’ reluctance to have direct conversations with their female peers, males admitted that if their romantic and/or sexual partners had a strong preference regarding VMMC, this influenced their decision. Male adolescents in relationships appeared to include partners in the decision-making process. One adolescent reported that his girlfriend’s persistence in persuading him to get circumcised was a major reason he ultimately underwent VMMC.

She [my girlfriend] used to nag me every day and told me that I needed to get circumcised … she told me that I have made the right decision, and our love has blossomed even more. (Male, age 18, Mbeya, Tanzania)

While only a few males reported their girlfriends directly influenced their decision to undergo VMMC, others mentioned such encouragement as a motivating factor.

#### Females Leveraging Relationship Status on VMMC

Young females admitted they both covertly and overtly tried to influence their partners’ decision to seek VMMC. Most female participants in Tanzania and Zimbabwe disclosed they would not initiate relationships or would readily discontinue them if their partners refused VMMC. While a few female participants mentioned using the threat of infections, HIV, and cervical/penile cancer as a means of persuasion, others believed that, regardless of benefits for herself, if a female truly cares for her partner, it is her duty to convince him to seek VMMC.

I would encourage [my boyfriend]. I would use different ways [to encourage him], ask him how he feels about circumcision … then ask him if he thinks it’s better he goes [and gets circumcised], so that once we decide to have sex, we know that we are well protected, we don’t have stress, it’s just the two of us. (Female, age 17, Orange Farm, South Africa)

Some females disclosed they used the power of maintaining the relationship as leverage:

If a male knows his girlfriend loves him, and she tells him to do anything, he would listen to her. As for me, if he does not accept to go and get circumcised, I would leave him for the one who has. He must listen and accept to be circumcised if he wants to keep the relationship. (Female, age 19, Mbeya, Tanzania)

#### Sexual Intimacy as an Incentive for VMMC

Overall, male adolescents noted that female preference for dating or having sex with circumcised males is a factor in their VMMC decision making.

I have heard some girls saying, ‘We now want guys who are circumcised, we no longer want the uncircumcised ones as they may have *chirwere* (HIV/AIDS).’ (Male, age 16, Mount Darwin, Zimbabwe)

Many female adolescents mentioned that having a circumcised male partner not only means feeling protected from HIV transmission, but appears more hygienic and sexually appealing. Females also mentioned that sex was more pleasurable with a circumcised as compared to an uncircumcised partner because of a belief that a circumcised male can prolong sex.

He will not come too early [when circumcised]; he will do so after some time has elapsed. It helps you to also enjoy [sex] as a woman. Because it is not nice when you agree to have sex, and the man comes just as he is getting in. (Female, age 18, Mount Darwin, Zimbabwe)

Likewise, male adolescents believed VMMC would enable them to sexually satisfy their female partner.

They [females] said it is not right as a man to have foreskin …they state [it] clear; they said when you have sex they don’t feel you right. (Male, age 18, Ermelo, South Africa)

#### Confidence/Perceived Attractiveness as a Byproduct

According to both genders, males seemed more confident overall following VMMC because they were more comfortable with their appearance. One young male reportedly hid the fact that he was not circumcised and felt scared a female would find out until he finally underwent VMMC:

To be sincere it was embarrassing [to not be circumcised], and I was feeling bad, because even when you want to urinate you will have to go to a place where nobody will see you. And also there was a certain feeling that makes you to be uncomfortable as a man. For instance, when you approach a girl, you cannot be straight to her [about your circumcision status] … you become scared. She can agree [to have sex] but yet you are scared … you run away. (Male, age 19, Iringa, Tanzania)

Gaining confidence when approaching partners for sexual encounters was a benefit reported by both males and females.

They [circumcised males] become more confident, because even when they are with a female partner they are more comfortable. A person cannot be comfortable with his partner if he is not circumcised. He could even prefer to have sex when the lights are off. After he is circumcised, he becomes free and comfortable. (Female, age 19, Mbeya, Tanzania)

At the same time, some adolescent females did not approve of this gain in confidence, since they felt that males become more promiscuous following the procedure.

I think what’s bad about circumcision is that those who have been circumcised tend to think they can’t be infected by any disease, and then such an individual will be forced to do what? To have sex with different people because he will be telling himself he won’t be infected because he is circumcised. That’s not true. (Female, age 19, Bulawayo, Zimbabwe)

### Female Support After VMMC

#### Males Feeling Supported by Females After VMMC

After undergoing VMMC, male adolescents felt supported by female peers, romantic partners, and sisters. Males reported females often approached them after the procedure, wanting to know details about pain, the procedure itself, and how it changed the appearance of the penis. For instance, one male talked about his sisters’ reaction:

My sisters know … they did not ask me a lot of questions about it because they know that I am a male and they are females, they just asked minor questions. They asked if I cried, and I told them I didn’t … I just told them I was circumcised … they told me it is well and I have become clean now … They laughed at me at the beginning, but at the end my elder sister told me I had made a good decision. (Male, age 18, Mbeya, Tanzania)

Males rarely reported being ridiculed or mocked by female peers for their decision to be circumcised, despite having this fear prior to the procedure. In Zimbabwe, one male noted that females were more cautious around recently circumcised males.

They just see [recently circumcised boys] as … they seem reluctant to play with them. They sit very far from them. They are afraid they might trigger something and you get hurt. They don’t want to come near someone who has been circumcised. (Male, age 14, Mount Darwin, Zimbabwe)

## DISCUSSION

This study demonstrates that adolescent female participants endorse and influence VMMC decision making by adolescent males. They reported leveraging their romantic relationships—or the potential for a relationship—to convince males to seek VMMC, and they remained supportive of the decision postprocedure. Adolescent males also viewed their romantic relationships as playing a role in VMMC, and they perceived females in their lives as supportive in their recovery. However, males reported being hesitant to discuss VMMC with female peers with whom they were not in romantic relationships. In general, adolescent females could be contributing to shaping social norms that encourage adolescent VMMC and that heighten stigma against those not seeking VMMC services [27].

Female adolescents’ discussions about VMMC in this study were consistent with research in Kenya, Uganda, Malawi, and South Africa, which showed that adult female partners’ opinions can have an influence on men’s decision to undergo VMMC [17, 18, 26, 29]. Women in Malawi and Uganda reported greater sexual satisfaction with circumcised partners as compared to uncircumcised partners, and women perceived circumcised men as more hygienic and carrying fewer diseases than uncircumcised men [19, 30]. A study of women in Kenya revealed that a man’s circumcision status was an important factor for determining whether to initiate sexual relations [18].

Females’ overall positive support of VMMC is also reflected in studies of the male perspective. For instance, a study of adolescent and adult men seeking VMMC in Uganda found that those who were in a relationship or were married had been influenced by their female partner to seek VMMC [29]. These men expressed concern for their relationships throughout the decision process and emphasized the need to involve women in awareness-raising activities. They viewed women as holding negotiating power when communicating with their male partners and being likely to persuade men to get circumcised, making it a joint decision [17, 18]. One media campaign in South Africa capitalized on adult women’s influence, with television ads showing women in a salon talking about how they convinced their partners to seek VMMC and how sexy they find the men once they do so [31]. Similar media campaigns may also be effective among adolescents.

Some female study participants expressed concern that VMMC is a driver of promiscuous behavior. Research on risk compensation after VMMC shows this concern is unfounded—VMMC is generally not associated with an increase in the number of sexual partners nor a reduction in future condom use [32–36]. Achieving greater buy-in from adolescent females for VMMC and encouraging them to play a role in convincing their male peers and romantic partners to seek VMMC services may require debunking this promiscuity myth.

This study has limitations. The findings are qualitative and therefore not generalizable beyond the included participants. It is possible that participants did not fully disclose personal details such as experience with circumcised vs uncircumcised males in the case of female participants, or male accounts of females ridiculing them for seeking or not seeking VMMC. To mitigate this, we triangulated data from FGDs of female adolescents and IDIs conducted with adolescent VMMC clients looking for corroborating and contradicting information.

While the decision to undergo VMMC is ultimately that of the adolescent and/or his parent/guardian, it is evident that adolescent females in this study used their position as current or potential partners, alongside the many benefits of VMMC (mutual partner health, attractiveness, sexual desire), as leveraging points for encouraging adolescent males to seek VMMC. Health programs should take the perspectives and influence of adolescent girls and young women into account as part of engaging adolescent male clients in VMMC and other HIV-related initiatives.

## Notes

***Acknowledgments.*** We are grateful to the adolescent participants in this study for sharing their perspectives and experiences. The authors also thank the VMMC facility managers, community mobilizers, and providers for their support, and acknowledge the contributions of the Technical Advisory Group for the adolescent VMMC assessment and their guidance throughout the study. We are privileged to work with this group of dedicated professionals from the US President’s Emergency Plan for AIDS Relief (PEPFAR), Centers for Disease Control and Prevention, Department of Defense, World Health Organization, United Nations Children’s Fund, and the Bill & Melinda Gates Foundation. Thanks go to the Human Sciences Research Council in South Africa, CSK Research Solutions in Tanzania, PSI/Zimbabwe, and the Centre for Sexual Health and HIV/AIDS Research in Zimbabwe for their assistance with data collection. The authors also appreciate the assistance of Meaghen Murphy with copy editing and Maria Elena Figueroa with project startup.

***Financial support.*** This work was supported by USAID with PEPFAR funding (cooperative agreement number AID-OAA-A-12-00058) to the Johns Hopkins Center for Communication Programs and was co-funded by the UK Department of International Development through the Integrated Support Program in Zimbabwe.

***Supplement sponsorship.*** This article appears as part of the supplement “Adolescent Voluntary Medical Male Circumcision: Vital Intervention Yet Improvements Needed,” sponsored by Johns Hopkins University.

***Potential conflicts of interest.*** All authors: No reported conflicts of interest. All authors have submitted the ICMJE Form for Disclosure of Potential Conflicts of Interest. Conflicts that the editors consider relevant to the content of the manuscript have been disclosed.
